# Harnessing Walnut-Based Zinc Oxide Nanoparticles: A Sustainable Approach to Combat the Disease Complex of *Meloidogyne arenaria* and *Macrophomina phaseolina* in Cowpea

**DOI:** 10.3390/plants13131743

**Published:** 2024-06-24

**Authors:** Mir Akhtar Hussain, Ghazala Parveen, Aashaq Hussain Bhat, Zubair Altaf Reshi, Farid S. Ataya, Zaffar A. Handoo

**Affiliations:** 1 Section of Plant Pathology and Nematology, Department of Botany, Aligarh Muslim University, Aligarh 202002, India; dr.ghazalaparveen.bot@gmail.com; 2 Department of Research Analytics, Saveetha Dental College and Hospitals, Saveetha Institute of Medical and Technical Sciences (SIMATS), Saveetha University, Chennai 600077, India; aashiqhussainbhat10@gmail.com; 3 Plant Biotechnology Laboratory, Department of Botany, Aligarh Muslim University, Aligarh 202002, India; zubairreshi26@gmail.com; 4 Department of Biochemistry, College of Science, King Saud University, P.O. Box 2455, Riyadh 11451, Saudi Arabia; fataya@ksu.edu.sa; 5 Mycology & Nematology Genetic Diversity & Biology Laboratory, USDA, ARS, Bldg. 010A, Rm. 111, 118, BARC-West 10300 Baltimore Avenue, Beltsville, MD 20705, USA; zafar.handoo@ars.usda.gov

**Keywords:** ZnO NPs, antifungal activity, nematicidal effect, oxidative stress mitigation, plant growth enhancement, histolocalization of ROS

## Abstract

Zinc oxide nanoparticles (ZnO NPs) exhibit diverse applications, including antimicrobial, UV-blocking, and catalytic properties, due to their unique structure and properties. This study focused on the characterization of zinc oxide nanoparticles (ZnO NPs) synthesized from Juglans regia leaves and their application in mitigating the impact of simultaneous infection by *Meloidogyne arenaria* (root-knot nematode) and *Macrophomina phaseolina* (root-rot fungus) in cowpea plants. The characterization of ZnO NPs was carried out through various analytical techniques, including UV–visible spectrophotometry, Powder-XRD analysis, FT-IR spectroscopy, and SEM-EDX analysis. The study confirmed the successful synthesis of ZnO NPs with a hexagonal wurtzite structure and exceptional purity. Under in vitro conditions, ZnO NPs exhibited significant nematicidal and antifungal activities. The mortality of *M. arenaria* juveniles increased with rising ZnO NP concentrations, and a similar trend was observed in the inhibition of *M. phaseolina* mycelial growth. SEM studies revealed physical damage to nematodes and structural distortions in fungal hyphae due to ZnO NP treatment. In infected cowpea plants, ZnO NPs significantly improved plant growth parameters, including plant length, fresh mass, and dry mass, especially at higher concentrations. Leghemoglobin content and the number of root nodules also increased after ZnO NP treatment. Additionally, ZnO NPs reduced gall formation and egg mass production by *M. arenaria* nematodes and effectively inhibited the growth of *M. phaseolina* in the roots. Furthermore, histochemical analyses demonstrated a reduction in oxidative stress, as indicated by decreased levels of reactive oxygen species (ROS) and lipid peroxidation in ZnO NP-treated plants. These findings highlight the potential of green-synthesized ZnO NPs as an eco-friendly and effective solution to manage disease complex in cowpea caused by simultaneous nematode and fungal infections.

## 1. Introduction

Plant pests and pathogens cause substantial damage to global crop production, resulting in estimated annual losses ranging from 20% to 40% [1]. Current pest management strategies predominantly rely on the widespread use of pesticides, including insecticides, fungicides, and herbicides. Despite their advantages, such as ready availability, rapid efficacy, and reliability, pesticides have detrimental repercussions, such as harm to non-target organisms, the resurgence of pest populations, and the emergence of resistance issues [1]. Moreover, it is concerning that approximately 90% of applied pesticides are lost during or after application, leading to direct environmental contamination. This contamination contributes to a 35% reduction in soil respiration, and nearly 90% of water sources in agricultural areas are tainted with pesticide residues. The consequences of these actions ripple through ecosystems, with bioaccumulation and biomagnification posing threats to aquatic and terrestrial food chains, as well as endangering natural biodiversity and pollinators [2]. Given these alarming ecological and agricultural challenges, there is an urgent need to explore innovative avenues for the development of cost-effective, high-performance pesticides. These new solutions should prioritize reduced environmental impact, safeguarding consumers and preserving food chains, and offer more precise control over pathogens, ultimately enhancing crop productivity.

Researchers have explored various eco-friendly strategies with potential nematicidal and antifungal activities. One significant measure that has emerged is the application of green-synthesized metal oxide-based nanoparticles [3,4]. Green synthesis can involve any plant part, including leaves, roots, flowers, stems, fruits, etc. [5]. Nanoparticles, engineered from metalloids, metallic oxides, nonmetals, and carbon nanomaterials within the size range of 1 to 100 nm, exhibit the potential to effectively combat plant diseases. Nanoparticles possess remarkable properties, including enhanced reactivity, unique optical, mechanical, electrical, and thermal characteristics, as well as high surface area-to-volume ratios. These properties make nanoparticles versatile in applications spanning catalysis, electronics, materials science, energy, medicine, cosmetics, and environmental remediation [6]. Among the various metal oxide nanoparticles, zinc oxide nanoparticles (ZnO NPs) represent a highly engineered and extensively employed option. They are synthesized using both chemical and green synthesis methods and have found extensive applications in plant disease management, serving as nanofungicides and constituents of nanobioformulations. The popularity of ZnO NPs can be attributed to their unique attributes, including precise targeting capabilities, heightened bioavailability owing to increased solubility and permeability, reduced required dosages, minimized dose-dependent toxicity, and controlled release mechanisms [7]. Several researchers have studied the antifungal activity of ZnO NPs synthesized through green synthesis [8,9,10], and it has become evident that ZnO NPs possess potential antifungal and nematicidal properties [11,12,13].

Walnut (*Juglans regia* L.) has a long history of medicinal and therapeutic uses, including antiseptic, anti-inflammatory, antidiabetic, and anti-helminthic properties. Recent research has explored the plant’s efficacy against fungal, bacterial, and plant parasitic nematode diseases [14,15,16,17]. Juglans regia leaves are rich in secondary metabolites such as phenolic acids, flavonoids, organic acids, tocopherols, triterpenic acids, terpenes, terpenoids, tetralone derivatives, megastigmane derivatives, and hydroxy-1,4-naphthoquinone (juglone) derivatives, contributing to their therapeutic effects [18].

Cowpea (*Vigna unguiculata* L. Walp.) stands as a vital legume crop cultivated in tropical and subtropical regions worldwide. Notably, cowpea boasts an exceptional nutrient profile, serving as a rich protein source for human consumption, nutritious fodder for livestock, and a contributor to atmospheric nitrogen fixation [19]. Globally, approximately 6.5 million metric tons of cowpea are produced annually, covering 14.5 million hectares of agricultural land [20]. In India, cowpea cultivation yields approximately 2.21 million tons across 3.9 million hectares, with a national productivity rate averaging 683 kg per hectare [21].

Plant parasitic nematodes pose a significant threat to global crop productivity, leading to substantial reductions in agricultural yields. Among these nematodes, root-knot nematodes (*Meloidogyne* spp.) are especially destructive, directly contributing to extensive crop field damage and consequent food scarcity for consumers [22]. Cowpea, a commonly cultivated crop, is susceptible to these root-knot nematodes (RKN), which are major plant-parasitic pests with a worldwide presence [23]. Furthermore, cowpea has been identified as a significant host for *M. arenaria* in various regions, further underscoring the severity of this issue in global agriculture [24].

*Macrophomina phaseolina*, an extensively adaptable soil-borne fungal pathogen, has emerged as a significant threat to cowpea [20]. This pathogen is responsible for the development of charcoal rot, a devastating disease in cowpea that leads to complete yield loss, making it one of the most critical crop diseases [25]. Additionally, *M. phaseolina* contributes to disease complexes in pulse crops, often in conjunction with root-knot nematodes, resulting in substantial reductions in productivity across these agricultural systems [26].

This study endeavors to investigate an innovative approach for mitigating the disease complex involving *M. arenaria* root-knot nematodes and *M. phaseolina* root-rot fungus in cowpea. We aim to achieve this by subjecting the plants to various concentrations of ZnO NPs biosynthesized from walnut leaves.

## 2. Results

### 2.1. Characterization of Root-Knot Nematode and Root-Rot Fungus

In the present study, a comprehensive characterization of *Meloidogyne* spp. was conducted, employing a combination of morphological and molecular techniques. An examination of adult females revealed distinct perineal patterns, providing valuable morphological insights. The overall morphology of the perineal pattern in females of *M. arenaria* is similar to other species within the genus. Therefore, to confirm the identification of the Indian strain of *M. arenaria*, molecular analyses were performed targeting the D2/D3 fragments of LSU (28S) rDNA. Through an NBlast analysis of the LSU rDNA sequences from the specimens, a remarkable 100% similarity was observed with previously deposited and published sequences of *M. arenaria* in the NCBI database. Furthermore, upon comparison of the LSU rDNA sequences of the strain with those available in GenBank, no discernible nucleotide differences were found.

To establish a broader context and explore the evolutionary relationships, phylogenetic analyses were conducted using partial sequences from the large subunit ribosomal DNA region, in conjunction with sequences from other *Meloidogyne* species nematodes (Figure S1). These phylogenetic analyses not only supported the morphological and molecular data but also enabled the clear differentiation of *M. arenaria* from other root-knot nematode (RKN) species. Remarkably, the Indian isolate formed a distinct monophyletic clade, closely aligned with previously described *M. arenaria* populations documented across various geographical locations.

**Figure 1:**
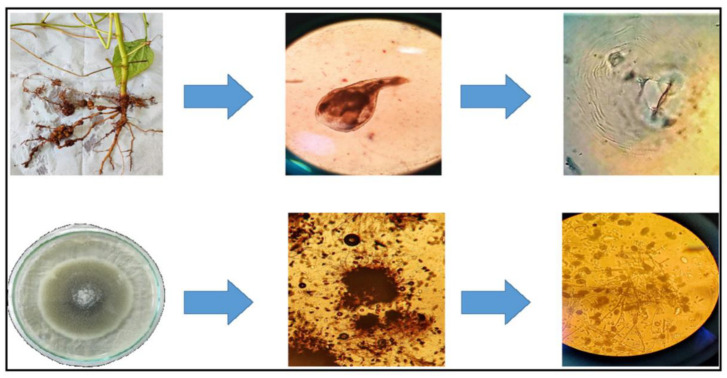
Collection and morphological identification of *M. arenaria* and *M. phaseolina*.

The morphological identification of root-rot fungus under a light microscope revealed distinct sclerotia of different sizes that resemble *Macrophomina phaseolina* (Figure 1). The molecular identification based on NBlast analysis of ITS rRNA confirmed that the pathogen showed 100% similarity with already deposited and published *M. phaseolina*, and the alignment of present sequences shows no discernible nucleotide differences with other deposited ITS rRNA sequences of *M. phaseolina* in the NCBI database. Phylogenetic analysis also showed that the present fungal stain formed a monophyletic clade with already described *M. phaseolina* populations from other geographical regions, hence described as the same (Figure S2).

### 2.2. Characterization of ZnO NPs and Their In Vitro Effects

#### 2.2.1. UV–Visible Analysis

**Figure 2:**
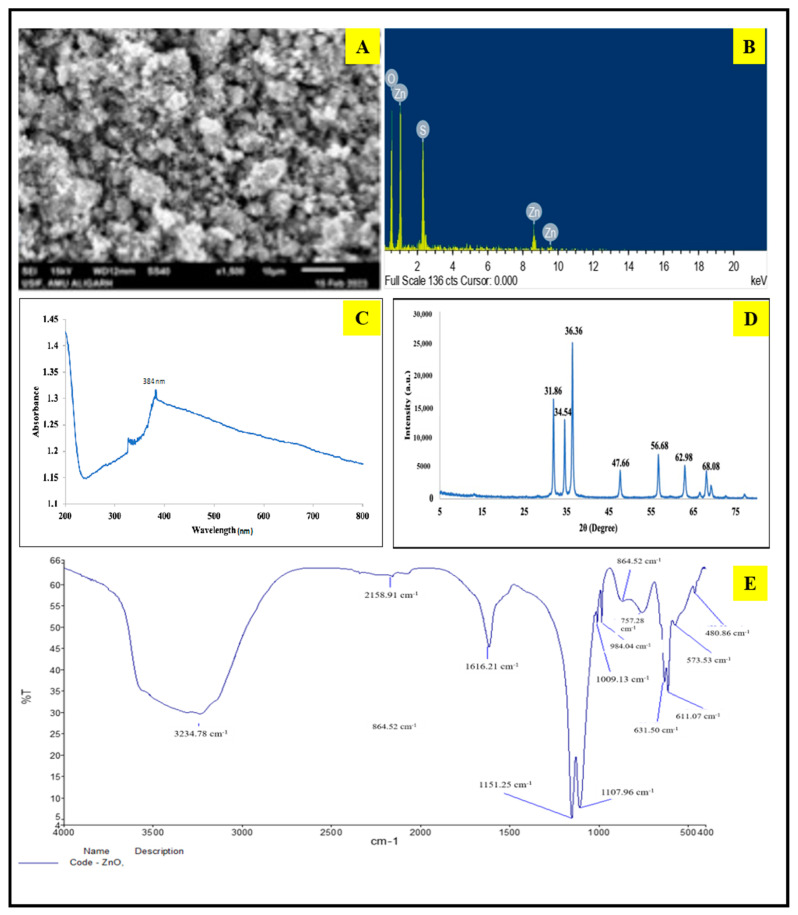
Characterization of walnut-based ZnO NPs. (**A**). SEM Micrograph. (**B**). EDX graphical pattern. (**C**). UV–vis absorption spectrum. (**D**). XRD pattern. (**E**). FT-IR spectrum.

The confirmation of the ZnO NP formation derived from Juglans regia leaves was achieved through UV–visible spectrophotometry Figure 2C), which investigated their optical properties. The spectrum depicted in Figure 2C represents the generated ZnO nanoparticles. The analysis of the UV–visible absorption spectrum, measured across a wavelength range of 200–800 nm, revealed a prominent absorbance peak at 384 nm. This peak corresponds to the characteristic band indicative of ZnO NPs.

#### 2.2.2. Powder-XRD Analysis

The Powder-XRD (PXRD) spectrum analysis of the synthesized ZnO nanoparticles revealed distinct peaks at 31.86°, 34.54°, 36.36°, 47.66°, 56.68°, 62.98°, and 68.06°. These peaks were well defined and narrow, and they were found to perfectly match a hexagonal wurtzite structure, in accordance with the established ICDD data (card number: 080–0075). This confirmation of the absence of impurities in the diffraction peaks serves as strong evidence for the exceptional purity and structural integrity of the resulting products (Figure 2D).

#### 2.2.3. FT-IR

In the FT-IR spectrum, ZnO NPs derived from walnut leaves exhibited distinctive peaks at 3234, 1616, and 1107 cm^−1^, corresponding to the stretching frequencies of ν(OH), ν(C=O), and ν(C=C), respectively. Moreover, a weak absorbance band observed at 480 cm^−1^ was attributed to ν(Zn-O) (Figure 2E).

#### 2.2.4. SEM-EDX

The surface characteristics and topography of the biosynthesized ZnO NPs analyzed through SEM are presented in Figure 2A. The SEM images vividly showcase the uniform dispersion and spherical arrangement of the ZnO NPs. Notably, these particles exhibited an average size of 65 nm. The frequency distribution histogram illustrating the size distribution of the ZnO NPs is presented in Figure 2B. Furthermore, the EDX histogram provides insight into the elemental composition of the ZnO nanoparticles, which were synthesized using the green synthesis method.

### 2.3. Effect of ZnO NPs on M. arenaria (J2) Mortality and M. phaseolina Growth under In Vitro Conditions

After treating *M. arenaria* J2 with ZnO NPs, the percentage of mortality in *M. arenaria* (J2) increased as the ZnO NP concentration increased. The J2 in distilled water showed a minimum mortality of 2.58%, while those subjected to ZnO NP treatments displayed mortality rates of 6.32%, 7.87%, 39.23%, 71.22%, 93.92%, and 94.12% at ZnO NP concentrations of 10, 20, 40, 60, 80, and 100 mgL^−1^, respectively (Table 1).

**Figure 3:**
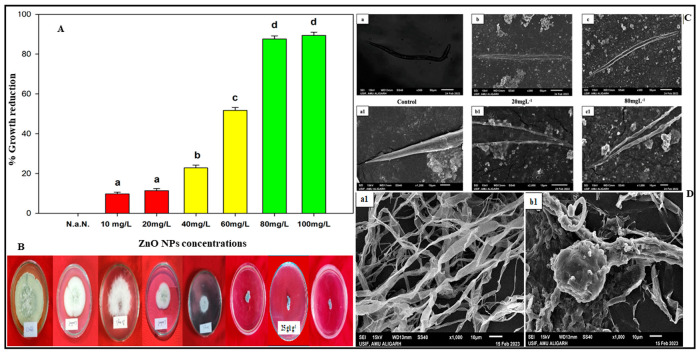
(**A**,**B**) Effect of ZnO NPs on percent growth reduction of *M. phaseolina* in vitro. (**C**,**a**,**a1**) Scanning electron micrographs (SEMs) of the second-stage juveniles (J2s) of *M. arenaria* in distilled water showing normal and smooth cuticle surface. (**C**,**b**,**b1**) SEM of the second-stage juveniles (J2s) of *M. arenaria* after exposure to ZnO NPs at low concentration showing minimum cuticle damage. (**C**,**c**,**c1**) SEM of the second-stage juveniles (J2s) of *M. arenaria* showing cuticle damage and corrugated surface. (**D**,**a1**) Untreated fungal mycelium of *M. phaseolina* showing normal and smooth hyphae. (**D**,**b1**) ZnO NPs treated fungal mycelium of *M. phaseolina* showing damaged, disturbed, and bulged hyphae.

Similarly, the in vitro studies of the fungal mycelial growth of *M. phaseolina* exposed to ZnO NP also demonstrated an increasing percentage of growth inhibition with rising concentration. The results indicated a significant (*p* ≤ 0.05) and maximum mycelial growth inhibition of 89.4% at 100 mgL^−1^, followed by 87.6% at 80 mgL^−1^ of ZnO NPs. At ZnO NP concentrations of 10, 20, 40, and 60 mgL^−1^, the growth inhibition percentages were 9.7%, 11.4%, 22.8%, and 51.7%, respectively (Figure 3A,B).

#### SEM Studies

The nematicidal effect of ZnO NPs was confirmed through SEM studies of *M. arenaria* J2. It was evident that the J2 treated with ZnO NPs suffered damage and shrinkage. In Figure 2C, untreated J2 appeared normal, exhibiting no physical damage. In contrast, the treated J2 exhibited distinct changes with a shrunken, wrinkled, and corrugated cuticle.

SEM studies were conducted on both untreated and ZnO NP-treated fungal mycelia of *M. phaseolina*, revealing a noticeable difference in their morphology (Figure 3D). The untreated M. phaseolina mycelium displayed typical reticulated hyphae with a smooth surface, as depicted in Figure 3D. However, following treatment with ZnO NPs, significant bulges appeared on the hyphal surface, accompanied by a loss of smooth morphology. This alteration indicates that the presence of ZnO NPs hindered the growth of *M. phaseolina* by inducing structural distortions in the hyphal architecture.

### 2.4. Effect of Biosynthesized ZnO NPs on Infected Cowpea Plants

#### 2.4.1. Effect on Plant Growth Parameters

Plants inoculated with nematodes and fungus showed a significant reduction of 50.72% in plant length compared to the untreated uninoculated control (UUC) (*p* ≤ 0.05). However, after treatment with ZnO NPs, the overall plant length improved with increasing the ZnO NP concentration up to 100 mgL^−1^. Similarly, the plant fresh weight of untreated inoculated plants (UIC) showed a 51.41% reduction compared to UUC plants. Nevertheless, the treatment of inoculated plants with ZnO NPs increased plant fresh weight, thereby mitigating the ill effects caused by the disease complex. Additionally, a significant improvement in plant dry mass was observed in infected plants treated with ZnO NPs compared to UIC (Table 2). Data analysis of all the growth parameters shows improvement with an increase in ZnO NP concentration. Although ZnO NP concentrations of 10, 20, and 40 mgL^−1^ did not yield significant results in plant growth, a very strong and significant improvement in plant growth was observed at concentrations of 60, 80, and 100 mgL^−1^.

#### 2.4.2. Effect on Leghemoglobin and Root Nodules

The LgHb content of untreated inoculated cowpea plants substantially decreased by 66.04% compared to UUC plants. However, after treatment with ZnO NPs, the LgHb content of plants returned to normal, with significant improvement observed at 100 mgL^−1^ followed by 80 mgL^−1^ (Table 2). Similarly, the number of root nodules significantly decreased in untreated inoculated plants by 70.58% compared to UUC. However, after treatment with ZnO NPs, the nodule number of roots increased, and a significant improvement in root nodule number was recorded in plants treated with ZnO NP concentrations of 80 and 100 mgL^−1^, respectively (Table 2). Meanwhile, it was observed that ZnO NP concentrations of 10, 20, and 40 mgL^−1^ were insignificant in improving the LgHb and nodule number of infected plants.

#### 2.4.3. Nematicidal and Antifungal Effect

**Figure 4:**
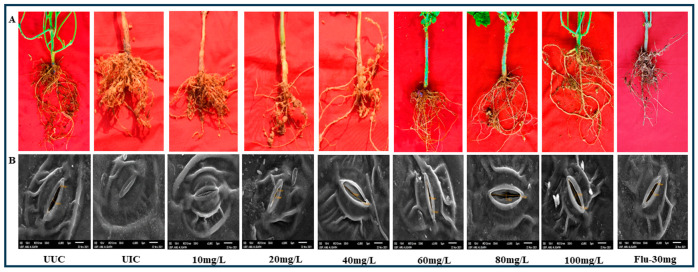
(**A**) Effect of ZnO NPs on the number of galls produced in cowpea infected with disease complex of *M. arenaria* and *M. phaseolina*. (**B**). Effect of ZnO NPs on the stomatal morphology in cowpea leaves infected with disease complex of *M. arenaria* and *M. phaseolina*.

ZnO NPs exhibited a very substantial reduction in both the multiplication of *M. arenaria* and the occurrence of galling in cowpea plants concurrently infected with *M. arenaria* nematodes and *M. phaseolina* root-rot fungus. In untreated, inoculated plants, the mean number of galls was recorded as 62.77. However, in plants treated with ZnO NPs, the number of galls decreased significantly in the plants treated with ZnO NPs (Figure 4A). The lowest number of galls (3.01) was observed in plants treated with ZnO NPs at concentrations of 100 mgL^−1^, followed by those treated with 80 mgL^−1^ (12.18) (Table 2). Additionally, the mean number of egg masses also exhibited a significant decline (*p* ≤ 0.05) when treated with 100 mgL^−1^ (4.03) and 80 mgL^−1^ (15.04) of ZnO NPs. The reproductive capacity of *M. arenaria* in infected cowpea plants was significantly reduced at higher ZnO NP doses compared to lower doses and untreated, inoculated plants.

Similarly, the antifungal activity of ZnO NPs at various doses in infected cowpea plants was assessed by examining the root-rot index. The lower doses of 10 mgL^−1^ and 20 mgL^−1^ of ZnO NPs did not exhibit a significant effect against *M. phaseolina*, resulting in a root-rot index of five for each dose, which was consistent with the untreated infected plants. However, the root-rot index decreased as the ZnO NP concentration increased. The lowest root-rot index of one was observed in plants treated with a ZnO NP concentration of 100 mgL^−1^, followed by those treated with 80 mgL^−1^ (2) and 60 mgL^−1^ (3) (Table 2).

### 2.5. Histochemical Localization and Quantification of ROS and Lipid Peroxidation

#### 2.5.1. Localization and Quantification of Superoxide Anion (O_2_^−^)

**Figure 5:**
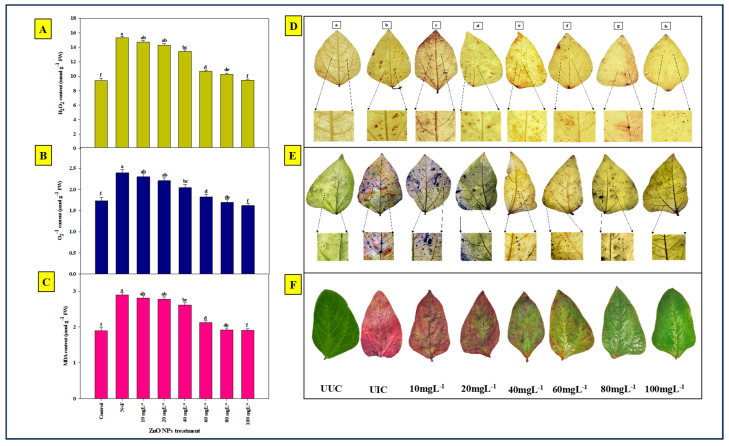
(**A**,**D**) Histochemical localization and estimation of H_2_O_2_ in UUC, UIC, and different ZnO NPs-treated leaves. (**B**,**E**). Histochemical localization and estimation of O_2_^−^ in UUC, UIC, and different ZnO NPs-treated leaves. (**C**,**F**). Histochemical localization and estimation of MDA in UUC, UIC, and different ZnO NPs-treated leaves.

Staining with NBT in the leaves of plants inoculated with root-knot nematode *M. arenaria* and root-rot fungus *M. phaseolina* revealed distinct blue colored dots. UUC plant leaves were used as a control. The results indicated that the highest accumulation of O_2_^−^ occurred in the UIC plants (2.40 µmolg^−1^ of fresh weight), while the lowest O_2_^−^ accumulation was observed in plants treated with ZnO NPs at a concentration 100 mgL^−1^ (1.62 µmolg^−1^), followed by 80 mgL^−1^ (1.70 µmolg^−1^) (Figure 5B,E). The accumulation of free radicals (O_2_^−^) on the leaves was represented by the presence of blue dots. The number and intensity of these blue dots decreased in a dose-dependent manner, with the fewest dots observed in plants treated with ZnO NP at 100 mgL^−1^ and the most in UIC plants. No blue dots were observed on UUC plant leaves.

#### 2.5.2. Localization and Quantification of Hydrogen Peroxide (H_2_O_2_)

Staining with DAB in the leaves of plants inoculated with root-knot nematode *M. arenaria* and root-rot fungus *M. phaseolina* revealed distinct brown colored dots. UUC plant leaves were used as the control. The results indicated that the highest accumulation of H_2_O_2_ occurred in the UIC plants (15.28 µmolg^−1^), while the lowest H_2_O_2_ accumulation was observed in plants treated with ZnO NPs at a concentration of 100 mgL^−1^ (9.44 µmolg^−1^), followed by 80 mgL^−1^ (10.26 µmolg^−1^) (Figure 5A,D). The accumulation of H_2_O_2_ on the leaves was represented by the presence of brown dots. Similar to O_2_^−^, the number and intensity of these brown dots decreased in a dose-dependent manner with the fewest dots observed in plants treated with ZnO NPs at 100 mgL^−1^, and the most in UIC plants. No brown dots were observed on UUC plant leaves.

#### 2.5.3. Oxidative Lipid Stress

To assess lipid peroxidation, Schiff’s reagent was used to visualize the peroxidation of membrane lipids. As shown in Figure 5F, the intensity of pink color increased as the concentration of ZnO NPs decreased. The lowest intensity of pink color was observed in plant leaves treated with ZnO NPs at a concentration of 100 mgL^−1^, followed by 80 mgL^−1^. Conversely, the highest intensity of pink color was observed in UIC leaves, followed by plants treated with ZnO NP at 10 mgL^−1^ and 20 mgL^−1^. A higher intensity of pink color indicates greater lipid peroxidation and consequently more disease symptoms. The highest MDA content was found in UIC plants (2.9 µmolg^−1^), while the lowest was in plants treated with ZnO NP at 100 mgL^−1^ (1.91 µmolg^−1^) (Figure 5C,F). No pink color was observed in UUC plant leaves.

### 2.6. SEM and Confocal Microscopy

Cowpea leaves were subjected to scanning electron microscopy to study the comparative changes in stomatal morphology in the leaves of UUC, UIC, and plants treated with different concentrations of ZnO NPs. The stress caused by the disease complex of *M. arenaria* and *M. phaseolina* in cowpea led to the total closure of the stomata. However, this was successfully managed by the application of ZnO NPs. In Figure 4B, the UIC plants showed completely closed stomata, whereas the stomata of cowpea plants treated with ZnO NPs at 80 mg/L and above showed fully opened and restored morphology, similar to the stomata of UUC plants. The stomata of plants treated with lower concentrations of ZnO NPs were partially opened. This indicates that ZnO NPs have a protective effect, helping cowpea plants maintain proper stomatal function under the dual stress conditions imposed by these pathogens. The restoration of stomatal integrity is crucial for the plant’s ability to regulate gas exchange and water loss, thereby enhancing its overall resilience and health.

**Figure 6:**
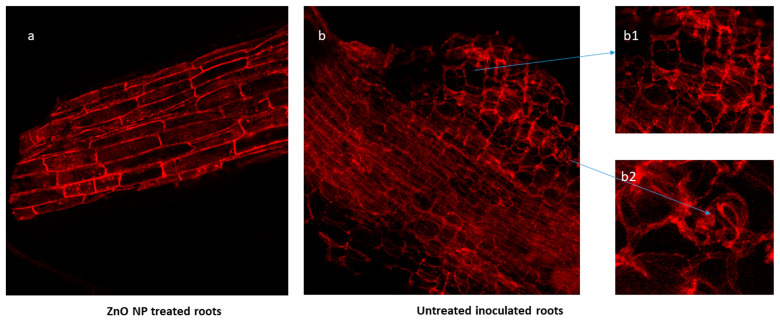
(**a**) Root section of ZnO NP-treated cowpea plant showing normal root anatomy. (**b**) Root section of untreated infected cowpea root showing (**b1**) giant cells with hypertrophy and hyperplasia (**b2**) presence of second-stage *M. arenaria* juveniles in root tissues.

The comparative studies of the fluorescent confocal micrograph of the fine roots of cowpea treated with 80 mg/L of ZnO NPs showed normal tissue morphology, and no root bulging and hypertrophy was seen (Figure 6a). However, the root section of the untreated inoculated control showed the formation of giant cells, feeding sites, hypertrophy, and hyperplasia which caused the root to bulge out, as shown in Figure 6b. This cellular disruption interferes with the plant’s vascular functions, severely affecting its overall health and growth. However, in our current experiment, the application of ZnO NPs successfully prevented these histological damages. This protective effect was clearly evident when comparing treated and untreated root sections through fluorescent confocal microscopy, as illustrated in Figure 6. The treated roots maintained normal cellular structure and vascular functionality, highlighting the potential of ZnO NPs in mitigating nematode-induced damage in plants.

## 3. Discussion

The current study aimed to characterize and assess the effects of walnut-based green-synthesized ZnO NPs on cowpea plants that were artificially inoculated with the root-knot nematode, *M. arenaria*, and the root-rot fungus, *M. phaseolina*, concurrently. Although the antifungal and nematicidal activity of both chemically and biosynthesized ZnO nanoparticles has already been studied by various researchers [13,27,28,29], the objective of this study is to promote environmentally friendly approaches that use fewer chemicals, are cost-effective, and manage diseases without harming the environment [30]. In the current study, various characterization procedures were applied to validate the purity and authenticity of the synthesized ZnO NPs. UV–visible spectrophotometry was used to confirm the optical properties and size of the nanoparticles, revealing a noticeable absorbance peak at 384 nm. The absorption peak at a shorter wavelength confirms that the particle size has become smaller, consistent with studies conducted by Darvishi et al. [31]. XRD patterns confirmed the crystalline nature of the nanostructure of green-synthesized ZnO NPs, in line with previous studies [31,32,33].

FT-IR spectra confirmed the presence of several functional groups attached to the surface of the ZnO NPs. Walnut leaves extract contains a significant amount of phenolic and aromatic compounds [31,34], including the characteristic naphthoquinone derivative compound Juglone, which imparts antifungal and nematicidal properties to ZnO NPs that are potentially effective against the disease complex of *M. arenaria* (root-knot nematode) [14,35] and *M. phaseolina* (root-rot fungus) [36,37,38] in cowpea. The FT-IR peaks resembled those obtained [39]. SEM-EDX analysis provided insights into the physical shape and structure of the green-synthesized ZnO NPs, and the elemental spectra confirmed the presence of elements other than zinc and oxygen, confirming their formation through green synthesis. This study aligns with SEM studies conducted by Siddiqui et al. [40].

In vitro studies demonstrated that ZnO NPs caused the mortality of J2 nematodes, consistent with studies conducted by Ahamed et al. [41]. The pot experiment results also showed a reduction in the reproduction factor with increasing ZnO NP concentration. Parameters such as the number of galls and egg masses were reduced when infected plants were treated with ZnO NPs. The potential harm to nematodes caused by ZnO NPs could result from various actions, including interference with cellular functions such as membrane permeability, ATP production, and triggering oxidative stress responses [42,43]. Additionally, the distribution pattern of ZnO NPs indicates a specific affinity for the nematode’s intestinal tissue as the primary target [44]. Juglone, as studied by some scientists earlier, could be one of the toxic components in walnut-based ZnO NPs for their nematicidal behavior [14,35]. Furthermore, the observed irregularities in J2 morphology through SEM imaging suggest that ZnO NPs could potentially disrupt the hypodermis and cuticle of nematodes by influencing components such as lipids, glycogen, and mucopolysaccharides [45].

Root-knot nematodes form giant cells and undergo hypertrophy and hyperplasia in the cortical area of root tissues, thereby disrupting the vascular functioning of the plants. However, the application of ZnO NPs prevented such histological damage, as observed in the current experiment through fluorescent confocal microscopy of treated and untreated root sections (Figure 5).

This study is in accordance with studies conducted by Lipovsky et al. [46] on beetroot. The antifungal activity of ZnO NPs was evident in their pronounced efficacy against *M. phaseolina* when cultured on PDA medium. Detailed examination using SEM unveiled discernible morphological alterations in the fungal mycelium due to the interaction with ZnO NPs, leading to the distortion, abnormal bulging, and damage of hyphal structures. This phenomenon is reminiscent of the deformations observed in hyphal growth and spore development, ultimately culminating in hyphal degeneration and cell demise, as documented by Ahamed et al. [41]. Additionally, this was supported by the pot experiment when infected cowpea plants were treated with ZnO NPs at different doses, resulting in a decrease in the root-rot index.

The concomitant infection of *M. arenaria* and *M. phaseolina* in cowpea significantly decreased the plant growth parameters, including plant length, plant fresh mass, and plant dry mass. However, with the treatment of ZnO NPs, plant growth improved and recovered back to normal at higher doses compared to the untreated, infected control (UUC). The plant growth recovery was a result of the mitigation of the concomitant stress caused by the root-rot fungus and root-knot nematode, resulting in the least incidence of infection in the plants treated with ZnO NPs at 80 mgL^−1^ and 100 mgL^−1^ concentrations. This study aligns with the research conducted by Maleita et al. [14] and Esteves et al. [34]. The concomitant stress of *M. arenaria* and *M. phaseolina* on cowpea also affected the stomatal morphology, and treatment with ZnO NPs restored its shape and aperture (Figure 4B). This observation is similar to the studies conducted earlier by Hussain et al. [47] on cowpea. Additionally, the number of nodules in the root system and hemoglobin content also increased with the application of ZnO NPs.

The antifungal activity of ZnO NPs is attributed to underlying mechanisms involving the generation of ROS and the release of Zn^2+^. These mechanisms selectively affect key components, such as N-acetyl glucosamine and β-1, 3-D-glucan synthase [48]. The production of ROS originates from the inherent properties of the NPs themselves, while the liberation of Zn^2+^ is a dissolution byproduct of the NPs within the culture medium [48]. This orchestrated ROS generation plays a crucial role in inducing oxidative stress, which can potentially hinder the function of antiproteases and simultaneously activate metalloproteases. This intricate interplay, as explained by Arciniegas-Grijalba et al. [49], subsequently triggers a cascade of events, including proteolysis and unchecked cellular degradation.

The application of walnut-based ZnO NPs was observed to mitigate the production of free radicals, including H_2_O_2_ and O_2_^−^, thus reducing oxidative stress. This observation was supported by histolocalization procedures applied to leaves when dipped in DAB and NBT, respectively. The intensity of the characteristic brown and blue colors became less prominent or vanished after NP application. The detoxification of plants from oxidative stress was further confirmed when the malondialdehyde (MDA) content decreased with the application of ZnO NPs, which aligns with the findings of Li et al. [50].

While the chemical and traditional methods of NP synthesis require the use of hazardous chemicals and high energy consumption, green synthesis offers a sustainable, cost-effective, and environmentally friendly alternative. In current studies, there was no apparent impact on the other microbiota living on or around the plants. One significant piece of evidence is that there was no decline in root-nodule formation with increasing NP concentration. Notably, there was no decline in the population of nitrogen-fixing bacteria, as the nodule numbers increased with concentrations ranging from 10 to 100 mg/L. This confirms that the nitrogen-fixing bacteria were not harmed throughout the entire application process. Thus, walnut-based green-synthesized ZnO nanoparticles offer a promising, environmentally friendly approach to managing nematode and fungal infections in cowpea plants. By harnessing the antifungal and nematicidal properties of naturally derived compounds, this study paves the way for sustainable agricultural practices that minimize chemical use and environmental impact, heralding a new era of eco-friendly disease management in crops.

## 4. Materials and Methods

### 4.1. Chemicals and Plant Material

The chemicals used in this study, including zinc acetate dehydrate (Zn(CH_3_COO)_2_·2H_2_O), NaOH, glutaraldehyde, absolute alcohol, methanol, and osmium tetroxide, were of analytical grade and were purchased from Sigma Aldrich (St. Louis, MO, USA). Streptomycin sulphate, an antibiotic, was obtained from the local certified drug store. Walnut leaves were collected from the Magam area of Jammu and Kashmir, India.

### 4.2. Preparation of Plant Leaf Extract

The walnut leaves were subjected to a cleansing process using dH_2_O, and then dried at room temperature. Afterward, the dried leaves were ground to a fine powder, and a mixture of 2 g of powdered leaves and 100 mL of dH_2_O was heated to 100 °C for 5 min. Following this, the resulting extract was filtered using Whatman filter paper no.1 and carefully stored at 4 °C.

### 4.3. Biosynthesis of ZnO NPs

For the biosynthesis of ZnO NPs, a 20 mL portion of the extract obtained from walnut leaves was heated to 50 °C for 10 min. This heated extract was then gradually added, drop by drop, to a 500 mL solution of zinc acetate dehydrate with concentrations ranging from 2 to 20 mM. The mixture was continuously stirred at 70 °C for approximately 3 to 4 h. As a result, the initially yellowish color of the reaction mixture turned into a creamy color, indicating the formation of zinc hydroxide. Subsequently, the cream-like zinc hydroxide precipitate was collected through centrifugation at 8000× *g* for 10 min at 4 °C. The precipitate was purified by washing twice using dH_2_O and then dried in an oven at 60 °C for 24 h. Finally, the pure ZnO NPs were obtained by heating the zinc hydroxide precipitate [31].

### 4.4. Characterization of ZnO NPs

ZnO NPs were characterized using various instruments, including a UV–visible spectrophotometer, scanning electron microscopy (JEOL JSM–JSM 6510, Tokyo, Japan) with energy dispersive X-ray analysis (SEM-EDX), Fourier-transform infrared (FT-IR) spectroscopy, Agilent (Santa Clara, CA, USA), and X-ray diffraction (XRD), Thermo Fisher (Waltham, MA, USA). The UV absorption spectra of the synthesized ZnO NPs were determined by a UV–visible spectrophotometer. The morphology, size, composition, and spot analysis for the validation of NPs were studied by SEM-EDX at the Aligarh Muslim University’s sophisticated instrumentation facility. FT-IR spectroscopy was conducted to explore the functional group sites on the NPs and determine the various phytochemical constituents involved in the synthesis and stabilization of the ZnO NPs. This was accomplished using an FT-IR spectrometer (Perkin Elmer spectrum Two) with the KBr disk technique [51]. The spectral range examined was 4000–400 cm^−1^ with a resolution of 4 cm^−1^. X-ray diffraction was employed to identify the crystal phase of the ZnO NPs. The scanning of ZnO NPs was conducted at a 2θ angle ranging from 20° to 80° with a scan speed of 1° min^−1^ and a step size of 0.02° [52,53].

### 4.5. Collection of Root-Rot Fungus, Nitrogen-Fixing Bacteria, and Root-Knot Nematode

The experiment was conducted in the Plant Pathology and Nematology section of the Department of Botany at Aligarh Muslim University. The root-rot fungus, *M. phaseolina*, was collected and isolated from the root-rot infected cowpea field in the Aligarh district of western Uttar Pradesh. The fungus was molecularly characterized using ITS rRNA markers using the protocol of Sebumpan et al. [54] and Khan et al. [55]. The collected fungus was sub-cultured on potato dextrose agar medium (PDA) for further studies. The *Bradyrhizobium* strain, a commercially available root-nodule bacteria, was sourced from a Quarsi Agriculture Farm in Aligarh, India, and cultured in yeast mannitol broth (YMB) in flasks. These flasks were shaken at 200 rpm and incubated at 28 °C in a rotary shaker for 5 to 7 days until they reached a turbid state and the logarithmic phase, with a cell density of approximately 1 × 10^9^ cells mL^−1^ [56]. The 5-day-old bacterial culture was then used for the inoculation process [57].

Root-knot nematode juveniles were isolated from cowpea roots maintained in a net house in the Plant Pathology and Nematology section of the Department of Botany at Aligarh Muslim University, Aligarh, India. Brown to black egg masses from the cowpea plant roots were carefully picked with forceps and transferred onto a sieve lined with a thin layer of tissue paper. Subsequently, the sieve, along with the eggs, was placed in a Petri dish filled with dH_2_O to facilitate hatching. Juveniles that hatched were collected from the Petri dish, along with the water, at 24 h intervals and transferred to a beaker. Fresh water was added to the Petri dishes after each collection, and this practice was repeated until the required number of juveniles were obtained [47]. The number of juveniles (J2) was determined using a counting dish and stereomicroscope [58]. The morphological characterization of root-knot nematodes was based on the perineal pattern of adult females (Figure 6) [59]. The nematodes were also characterized molecularly using LSU rRNA gene studies following the protocol described by Bhat et al. [60] and Bhat et al. [61]. Briefly, the DNA was extracted from single females using an extraction buffer. Single virgin females were individually transferred into sterile PCR tubes (0.2 mL) containing 20 μL of extraction buffer (17.6 μL of nuclease-free dH_2_O, 2 μL of 5X PCR buffer, 0.2 μL of 1% Tween, and 0.2 μL of proteinase K). Samples were frozen at –20 °C for 60 min and then immediately incubated in a PCR thermocycler at 65 °C for 1.2 h, followed by incubation at 95 °C for 10 min. The resulting supernatants were used as DNA templates to amplify D2D3 regions of the 28S rRNA using primers D2F (5′-CCTTAG TAACGGCGAGTGAAA-3′) (forward) and 536 (5′-CAGCTATCCTGAGGAAAC-3′) (reverse) [62]. The amplified products were Sanger sequenced and submitted to NCBI under an accession number and used for molecular and phylogenetic analysis.

### 4.6. In Vitro Studies

#### 4.6.1. Antifungal Activity of ZnO Nanoparticles

The in vitro antifungal activity of ZnO NPs derived from walnut leaves was evaluated using concentrations of 10 mgL^−1^, 20 mgL^−1^, 40 mgL^−1^, 60 mgL^−1^, 80 mgL^−1^, and 100 mgL^−1^, respectively, in PDA medium. Petri dishes with a size of 90 × 15 mm were fully autoclaved before use. An absolute control, containing PDA with 0 mgL^−1^ of ZnO NP, was maintained for comparison. Three replicates were used for each concentration, and all the readings were calculated as the mean of the three replicates.

The poisoned food in vitro technique, as described by Das et al. [63], was employed to assess the antifungal activity. ZnO NPs at the aforementioned concentrations were prepared and mixed with the PDA medium immediately before pouring it into the Petri dishes. The Petri dishes were then arranged and labeled according to the concentrations of NPs. To prevent bacterial growth, 1 mL of streptomycin sulphate with a strength of 100 ppm was added to the PDA medium just before pouring it into the Petri plates following the method of [64]. A fungal mycelial plug with a diameter of 5 mm was obtained from a pure culture of fungus *M. phaseolina* and inoculated into all the Petri dishes, including the untreated control [65]. The inoculated Petri dishes were sealed with paraffin film and incubated in a BOD at 27 ± 1 °C. The experiment comprised three replicates for each treatment, and the percentage of growth inhibition was calculated after 7 days of inoculation using the formula of [66,67]

$$Mycelial\;growth\;inhibition=\frac{Dc-Dt}{Dc\times 100}$$

where Dc (mm) = diameter of control set and Dt (mm) = diameter of treated set.

#### 4.6.2. Nematicidal Activity of ZnO Nanoparticles

The nematicidal efficacy against the second-stage juveniles (J2) of *Meloidogyne arenaria* was evaluated by testing various concentrations (10, 20, 40, 60, 80, and 100 mgL^−1^) of ZnO NPs biosynthesized from walnut leaves. To assess the impact of ZnO NPs on J2 mortality, 10 mL of dH_2_O containing 100 newly hatched J2s was placed in Petri plates along with 8.0 mL of the respective ZnO NP concentrations. Control Petri dishes containing only dH_2_O were utilized for comparison. To prevent evaporation, parafilm was applied to cover the Petri plates, and J2 mortality was observed after 48 h using a stereomicroscope. Each treatment was replicated five times. The J2s showing movement or twisting were considered alive [68], while those that remained motionless and straight, even after being exposed to tap water and pricked with a needle, were considered dead [69]. Probit analysis was employed to determine the LC50 value for each treatment based on the concentration and percent mortality data [13]. The formula for calculating percent mortality is as follows:

$$Percent\;mortality=\frac{Cc-Ct}{Cc\times 100}$$

where Cc  = count of active J2s in the control and Ct  =  number of active J2s after 48 h of exposure to various concentrations of biosynthesized ZnO NPs.

#### 4.6.3. Scanning Electron Microscopy of Root-Rot Fungi and Root-Knot Nematode

Scanning electron microscopy (SEM) was employed to investigate the impact of biosynthesized ZnO NPs on the root-rot fungus *M. phaseolina* and the root-knot nematode *M. arenaria*. Both the fungus and the nematodes were separately exposed to ZnO NPs at concentrations of 20 and 80 mg mL^−1^ for 7 days at ambient temperature. Untreated fungal and nematode samples were also maintained for comparison, following the experimental procedure outlined by Khan et al. [44]. After exposure to ZnO NPs, both the fungal and nematode samples were separately fixed in a 2.5% glutaraldehyde solution for 4 h and subjected to three rounds of washing with phosphate-buffered saline (PPB). The sequential dehydration of the samples was carried out using a graded ethanol series (10%, 20%, 40%, 60%, 80%, and 90%), with each step lasting 15 min at room temperature. Subsequently, the samples were immersed twice in 100% ethanol. They were then separately placed in hexamethyldisilazane (HMDS) for 5 min at room temperature. Afterward, the samples were dried in a desiccator for 30 min, fixed onto SEM stubs, sputter-coated with a layer of gold, and examined using the JSM-6510LV scanning electron microscope (JEOL, Tokyo, Japan).

### 4.7. Pot Experiment

#### 4.7.1. Experimental Design

The antifungal and nematicidal activity of ZnO NPs derived from walnut leaves was evaluated in a pot experiment using cowpea plants artificially infected with a disease complex consisting of root-knot nematodes (*M. arenaria*) and the root-rot fungus (*M. phaseolina*). The pot experiments were conducted in a greenhouse at the Department of Botany, Aligarh Muslim University (Aligarh, Uttar Pradesh, India) (27.914108° N, 78.072639° E, 187.45 masl). The experiments were carried out in a completely randomized design. Cycles of 25 °C/15 °C day/night with a relative humidity of 68% were maintained throughout the experiments. Cowpea seeds (Kashi Kanchan variety) were first sterilized in a 0.1% NaOCl solution for 5 min, followed by three rinses with sterilized water. Subsequently, the sterilized seeds were soaked in a suspension of ZnO NPs at concentrations of 10, 20, 40, 60, 80, and 100 mgL^−1^ under static conditions for 12 h. Unprimed seeds were kept as controls. The primed seeds and unprimed seeds were then separately sowed in well-sterilized, autoclaved, and labeled clay pots (25 cm in length and height) filled with a mixture of soil and farmyard manure in a 3:1 ratio. Five seeds were sown in each pot (25 cm in length and height). After germination, only one seed was maintained per pot. Plants were watered every other day. Ten days after planting, the seedlings were subjected to inoculation with both *M. phaseolina* and *M. arenaria* J2s per pot. To expose the roots for inoculation, the top layer of soil around the plants was carefully removed. A sterile pipette was used to evenly distribute 10 mL of a fungus suspension (containing 1 g of *M. phaseolina*) and 10 mL of a nematode suspension (containing 2000 *M. arenaria* J2s) around the root zone [47,70].

#### 4.7.2. Scanning Electron and Confocal Microscopy

SEM analysis of the leaves was conducted using the JEOL JSM-6510LV microscope at the University Sophisticated Instrumentation Facility (USIF), AMU, Aligarh. Stomatal behavior in the leaves inoculated with a combination of *M. arenaria* J2 and *M. phaseolina* was observed under SEM, following the procedure outlined by Mir et al. [71]. For analysis, five-leaf cuttings per plant were selected. The samples were air-dried, cut, and then fixed with 2% paraformaldehyde and 2.5% glutaraldehyde for 2 h. Subsequently, the samples were washed with 0.1 M of sodium cacodylate buffer (pH 7.3), and lipid fixation was achieved using 1% osmium tetroxide. The samples were further processed as discussed above. Finally, the images were recorded at an accelerating voltage of 15 KV and 3000× magnifications.

For confocal microscopy of cowpea, propidium iodide (PI) dye was prepared by dissolving 0.1 mg of dye per mL of acetone. One week after inoculation, roots were taken and cleaned by tap water. After staining with 0.5 μg/mL of PI in phosphate-buffered saline for 5 min, the roots were observed under the laser scanning confocal microscope at 519 nm wavelength [72].

#### 4.7.3. Quantification and Histochemical Localization of Reactive Oxygen Species (ROS) and Lipid Peroxidation

The evaluation of plant damage resulting from the concurrent infection of root-rot fungus and root-knot nematodes, in comparison to the control, involved assessing the generation of ROS, including superoxide anion (O_2_^−^) and hydrogen peroxide (H_2_O_2_). This was accomplished through the utilization of nitroblue tetrazolium chloride (NBT) and the 3,3-diaminobenzidine (DAB) staining techniques applied to leaves [47]. For histochemical analysis, we selected five leaves from each treatment group. The quantification of the O_2_^−^ and H_2_O_2_ levels was performed following the protocol outlined by Mir et al. [71]. To assess the degree of membrane lipid peroxidation, we measured the production of malondialdehyde (MDA) [49]. Additionally, the histochemical detection of lipid peroxidation was accomplished using Schiff’s reagent [71].

### 4.8. Data Recording

#### 4.8.1. Estimation of Plant Growth Parameters

After 60 days following nematode and fungus inoculation, the plants were removed from the soil, and their roots were thoroughly cleaned using tap water. Subsequently, the length, fresh weight, and dry weight of the plants were measured. To facilitate data interpretation, the reduction in plant growth was calculated as a percentage decrease for all growth parameters.

#### 4.8.2. Leghemoglobin Content and Root Nodules

The method outlined by LaRue and Child [73] was employed to measure the leghemoglobin (LgHb) content in the root nodules. Fresh root nodules weighing 100 mg were ground and homogenized in 0.3 mL of pre-chilled PBS buffer (Na_2_HPO_4_-NaH_2_PO_4_ buffer at 5 °C, pH = 6.8). Following centrifugation at 12,000× *g* for 15 min, the supernatant was analyzed using a spectrophotometer at wavelengths of 540 nm, 520 nm, and 560 nm. The concentration of LgHb was determined by comparing it to a protein standard using Bovine Hb. The number of root nodules per root system was counted just after uprooting the plant, followed by removing adhering soil and washing under tap water.

#### 4.8.3. Recording the Disease Parameters

To evaluate the disease incidence and severity resulting from the co-infection of *M. arenaria* and *M. phaseolina*, several parameters were measured. These parameters included the number of galls, egg masses, and root rot percentage. Galls were counted per root system, and egg masses were identified as brownish-black structures on the root surface. Root rot severity was assessed using a scale ranging from 0 to 5, which indicated the percentage of symptoms observed on the roots. Especially, the scale was defined as follows: 0 = No disease symptoms observed, 1 = Up to 12.5% symptoms on the root, 2 = 12.6% to 25% symptoms on the root, 3 = 25.1% to 37.5% symptoms on the root, 4 = 37.6% to 50% symptoms on the root, and 5 = More than 50% symptoms on the roots [44]. To determine the nematode population in the soil, a 200 g subsample of soil was processed using Cobb’s sifting and decanting method, in combination with Baermann’s funnel technique [74]. A nematode suspension was prepared and diluted to a volume of 100 mL. Nematodes were counted using counting plates, with three counts performed for each sample. The final nematode population per kg of soil was determined by averaging these three counts.

For the estimation of the root nematode population, 1 g of roots from each plant replicate was macerated in an electrically powered Waring blender for approximately 30 to 40 s. The macerate was collected and adjusted to a volume of 100 mL in a beaker. The nematodes in the obtained suspensions were counted using a stereomicroscope.

The reproduction factor was subsequently calculated based on the obtained data using the following formula:

$$Reproduction\;factor\;(RF)=\frac{Final\;nematode\;population}{Initial\;nematode\;population}$$

### 4.9. Statistical Analysis

Data collection and interpretation were based on the mean values obtained from three independent replicates (n = 3). To analyze the data for statistical significance, we employed the Duncan multiple range test (DMRT) and utilized Statistical Package for Social Sciences (SPSS) version 17.0 software. A significance level of *p* ≤ 0.05 was applied to determine statistical significance.

## 5. Conclusions

The study successfully characterized ZnO NPs synthesized from Juglans regia leaves, demonstrating their purity and structural integrity. The UV–visible analysis revealed a prominent absorbance peak at 384 nm, confirming the formation of ZnO nanoparticles. Powder-XRD analysis confirmed the hexagonal wurtzite structure of the nanoparticles. FT-IR spectra indicated characteristic functional groups, and SEM-EDX provided insights into their size and elemental composition. Furthermore, the study demonstrated the potent antifungal and nematicidal properties of these biosynthesized ZnO NPs. They caused a significant mortality of *M. arenaria* J2 and inhibited the growth of *M. phaseolina* in vitro. SEM studies revealed damage to J2 nematodes and structural distortions in fungal hyphae upon NP treatment. In infected cowpea plants, ZnO NPs improved plant growth parameters, increased leghemoglobin content, and promoted root nodule formation while significantly reducing nematode galling and fungal root-rot. Histochemical analysis indicated a reduction in ROS accumulation and lipid peroxidation in plant leaves treated with ZnO NPs. These findings underscore the potential of walnut-based ZnO NPs as an environmentally friendly approach for managing co-infections of root-knot nematodes and root-rot fungi in cowpea. The ability to mitigate oxidative stress and enhance plant growth makes these nanoparticles a promising tool for sustainable agriculture, contributing to improved crop health and yield. These results, together with their eco-friendly synthesis method, provide valuable insights for addressing complex plant diseases while minimizing environmental impact.

## Figures and Tables

**Table 1:** Percent mortality of *Meloidogyne arenaria* juveniles (J2s), 48 h after treatment with walnut-based ZnO NPs at different concentrations.

Treatment mgL^−1^	Total J2s	% Mortality 48 h
Control	100	2.58 ± 0.18 ^a^
10 mgL^−1^	100	6.32 ± 0.23 ^b^
20 mgL^−1^	100	7.87 ± 0.28 ^b^
40 mgL^−1^	100	39.23 ± 1.05 ^c^
60 mgL^−1^	100	71.22 ± 1.54 ^d^
80 mgL^−1^	100	93.92 ± 1.67 ^e^
100 mgL^−1^	100	94.12 ± 1.67 ^e^

These letters denote the degree of significance between the readings calculated using software SPSS version 17. Two different letters signify that two readings are significant to each other. Same letter denote non significant variation.

**Table 2:** Effect of different concentrations of walnut-based ZnO nanoparticles on disease complex of root-knot nematode *Meloidogyne arenaria* and root-rot fungus *Macrophomina phaseolina*, growth parameters, root nodules, and leghemoglobin in *Vigna unguiculata* L. walp.

Doses	No. of Galls	Number of Egg Masses	Reproduction Factor	Root Rot Index	Plant Length Root + Shoot (% Change)	Plant Fresh Mass (% Change)	Plant Dry Mass (% Change)	Leg Hemoglobin mg/g Nodules (% Change)	No. of Nodules (% Change)
UUC	0	0	0	0	77.56 ± 1.41 ^c^	84.66 ± 1.55 ^f^	18.07 ± 0.32 ^f^	4.80 ± 0.08 ^f^	78.12 ± 1.44 ^f^
UIC	62.77 ± 1.1 ^f^	69.33 ± 1.2 ^f^	5.69 ± 0.10 ^g^	5	38.22 ± 0.63 ^a^ −50.72	41.13 ± 0.74 ^a^ −51.41	5.24 ± 0.12 ^a^ −71.0	1.63 ± 0.02 ^a^ −66.04	22.98 ± 0.46 ^a^ −70.58
10 mgL^−1^	58.65 ± 1.0 ^e^	66.01 ± 1.1 ^f^	5.22 ± 0.09 ^f^	5	40.08 ± 0.70 ^a^ −48.32	42.68 ± 0.77 ^ab^ −49.58	5.75 ± 0.13 ^a^ −68.17	1.66 ± 0.02 ^a^ −65.41	25.76 ± 0.49 ^a^ −67.02
20 mgL^−1^	56.91 ± 1.0 ^e^	61.91 ± 1.1 ^e^	4.89 ± 0.08 ^e^	5	43.19 ± 0.73 ^a^ −44.31	44.77 ± 0.83 ^b^ −47.11	7.36 ± 0.14 ^b^ −59.26	1.69 ± 0.03 ^a^ −64.79	30.14 ± 0.58 ^b^ −61.42
40 mgL^−1^	42.12 ± 0.7 ^d^	51.87 ± 0.9 ^d^	3.22 ± 0.05 ^d^	4	51.40 ± 0.92 ^ab^ −33.72	49.98 ± 0.85 ^c^ −40.96	8.98 ± 0.15 ^c^ −50.30	2.41 ± 0.04 ^b^ −49.79	39.56 ± 0.75 ^c^ −49.35
60 mgL^−1^	33.02 ± 0.5 ^c^	37.67 ± 0.6 ^c^	2.81 ± 0.05 ^c^	3	58.22 ± 1.02 ^ab^ −24.93	56.11 ± 1.01 ^d^ −33.72	11.90 ± 0.21 ^d^ −34.14	2.80 ± 0.05 ^c^ −41.66	48.12 ± 0.82 ^d^ −38.40
80 mgL^−1^	12.18 ± 0.2 ^b^	15.04 ± 0.2 ^b^	1.02 ± 0.01 ^b^	2	74.09 ± 1.35 ^c^ −4.47	73.08 ± 1.31 ^e^ −13.67	15.04 ± 0.27 ^e^ −16.76	3.42 ± 0.06 ^d^ −28.75	63.89 ± 1.15 ^e^ −18.21
100 mgL^−1^	3.01 ± 0.1 ^a^	4.03 ± 0.1 ^a^	0.4 ± 0.01 ^a^	1	78.44 ± 1.47 ^c^ +1.13	83.90 ± 1.52 ^f^ −0.89	17.87 ± 0.30 ^f^ −1.10	4.63 ± 0.08 ^e^ −3.54	76.13 ± 1.36 ^f^ −2.54

These letters denote the degree of significance between the readings calculated using software SPSS version 17. Two different letters signify that two readings are significant to each other. Same letter denote non significant variation.

## Data Availability

The datasets during and/or analyzed during the current study are available from the corresponding author on reasonable request. The data are not publicly available due to [This study is the part of a research program having different set of objectives with each experiment interlinked directly or indirectly. Hence, open availability of the data may breach the other ongoing objectives of the project].

## Supplementary Materials

The following supporting information can be downloaded at: https://www.mdpi.com/article/10.3390/plants13131743/s1, Figure S1. Phylogenetic tree of the genus Meloidogyne based on LSU of rDNA. Numbers above branches represent bootstrap support values. GenBank numbers are shown along with the taxon names. Figure S2. Phylogenetic tree of the genus Macrophomina based on ITS rRNA. Numbers above branches represent bootstrap support values. GenBank numbers are shown along with the taxon names.
