# Supplementary material for: Harnessing Walnut-Based Zinc Oxide Nanoparticles: A Sustainable Approach to Combat the Disease Complex of Meloidogyne arenaria and Macrophomina phaseolina in Cowpea
Source: Plants (Basel). 2024 Jun 24;13(13):1743. doi: 10.3390/plants13131743 (PMC11244520; doi:10.3390/plants13131743)
Supplement: Supplementary file 1 [file plants-13-01743-s001.zip › plants-3049419-supplementary.pdf]

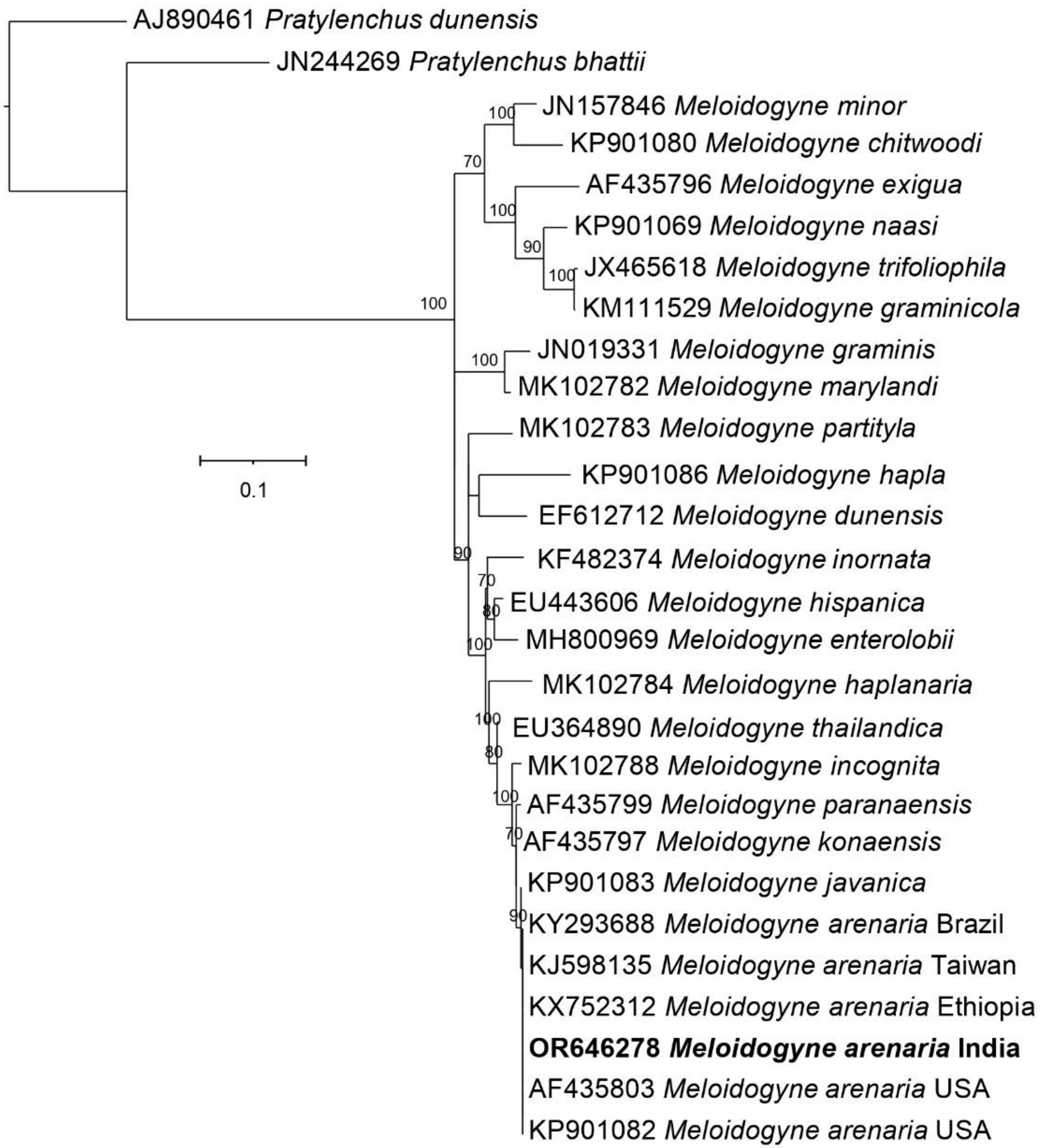

Figure S1. Phylogenetic tree of the genus *Meloidogyne* based on LSU of rDNA. Numbers above branches represent bootstrap support values. GenBank numbers are shown along with the taxon names.

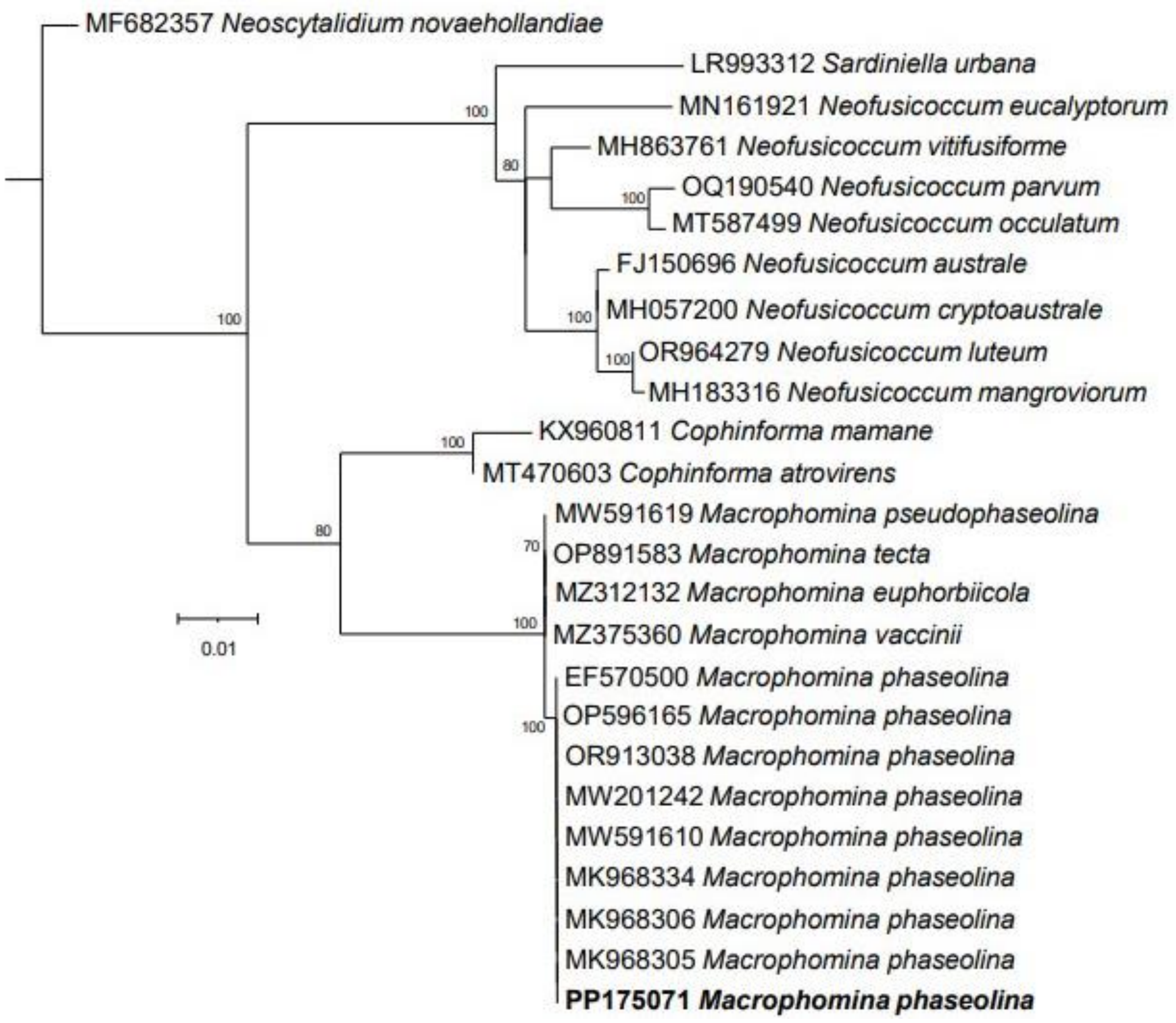

Figure S2. Phylogenetic tree of the genus *Macrophomina* based on ITS rRNA. Numbers above branches represent bootstrap support values. GenBank numbers are shown along with the taxon names.
